# *Jasonia glutinosa* (L.) DC.: Back in Our Pantries? A Review of Its Pharmacological Activity and Mechanisms of Action

**DOI:** 10.3390/ijms26062536

**Published:** 2025-03-12

**Authors:** Marta Sofía Valero, Carlota Gómez-Rincón, Víctor López, Francisco Les

**Affiliations:** 1 Departamento de Farmacología, Fisiología y Medicina Legal y Forense Facultad de Ciencias de la Salud, Universidad de Zaragoza, 50009 Zaragoza, Spain; 2 Instituto Agroalimentario de Aragón, IA2, Universidad de Zaragoza-CITA, 50009 Zaragoza, Spain; cgomez@usj.es (C.G.-R.); ilopez@usj.es (V.L.); 3 Departamento de Farmacia, Facultad de Ciencias de la Salud, Universidad San Jorge, 50830 Villanueva de Gállego, Spain

**Keywords:** biological activity, phytochemical, rock tea, traditional uses

## Abstract

*Jasonia glutinosa* (L.) DC., commonly known in Spain as “Rock Tea”, is a medicinal plant native to the Iberian Peninsula, southern France, and Morocco. It has traditionally been used as a digestive, analgesic, antimicrobial, antidepressant, or for respiratory diseases. This narrative review aims to scientifically validate the ethnopharmacological uses of *J. glutinosa* as a medicinal plant, emphasizing the relationship between its traditional applications, pharmacological activities, and mechanisms of action based on experimental evidence. A comprehensive search was conducted in various electronic databases to gather information on its traditional uses, phytochemical composition, and in vitro, ex vivo, and in vivo studies related to pharmacological properties. The literature review uncovered significant findings regarding the pharmacological and molecular mechanisms of this medicinal plant in various experimental models, particularly highlighting its spasmolytic, anti-inflammatory, and antioxidant properties.

## 1. Introduction

**Figure 1:**
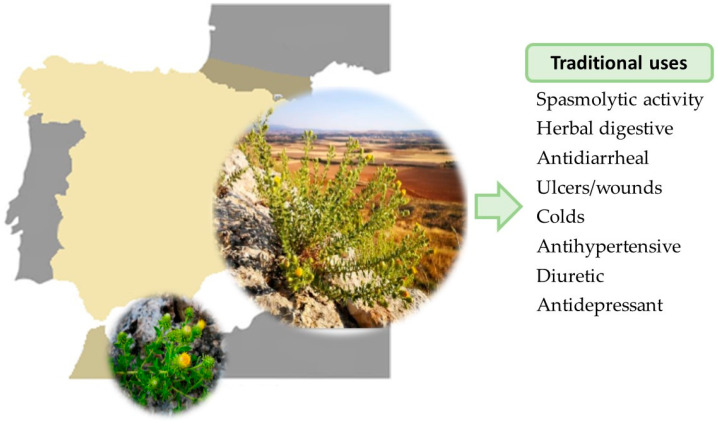
Distribution of *Jasonia glutinosa* (colored areas) and its traditional uses.

*Jasonia glutinosa* (L.) DC., a synonym of *Chiliadenus glutinosus* Fourr. and commonly known as rock tea, is an aromatic plant distributed throughout the western Mediterranean region, particularly in Spain, southern France, Malta, Sicily, and Morocco. It grows on limestone rocks, embedded in cracks, with no apparent pattern (Figure 1) [1]. Taxonomically, it belongs to the Astereaceae family. This perennial plant has woody roots and viscous stems, reaching a height of about 30–50 cm. Its small, lanceolate, glandular, and highly aromatic leaves are arranged alternately along the stem. The upper stem bears golden-yellow tubular flowers, which form clustered heads surrounded by glandular outer bracts. Flowering occurs between July and September [1,2].

*J. glutinosa* is a medicinal plant of significant relevance in the eastern regions of the Iberian Peninsula. For generations, the aerial parts, including the inflorescences, have been harvested at the onset of flowering and dried to prepare an infusion characterized by a camphor-like aroma and a bitter taste. This infusion is mainly used for its digestive properties, particularly as an eupeptic agent, and for the treatment of ulcers, spasms, diarrhea, nausea, and digestive discomfort such as gas or dyspepsia [1,2]. Beyond its gastrointestinal benefits, *J. glutinosa* has been traditionally used as an antihypertensive, a remedy for respiratory diseases, a diuretic, and a treatment for kidney ailments such as kidney stones and pain. Additionally, it has been employed for its antidepressant effects (Figure 1).

In recent years, interest in this plant has grown, particularly in traditional and complementary medicine. This rise in popularity is partly attributed to dissatisfaction with conventional healthcare services, chronic treatments, and the search for more natural substances for the prevention and treatment of pathologies. Furthermore, its organoleptic properties have attracted attention, leading to its incorporation into gastronomy. *J. glutinosa* is now commonly found in restaurants, where it is used in infusions, cold teas, ice creams, and as an ingredient in various desserts [2].

As a result of these factors, *J. glutinosa* is becoming increasingly available in the herbal stores and pharmacies. In fact, this plant is marketed by various companies and can be purchased through online platforms.

*J. glutinosa* is one of the most popular species of medicinal plants in the Iberian Peninsula. Due to growing interest in this plant, an increasing number of scientific studies aim to validate its traditional uses [1,2,3]. This review seeks to complement the existing knowledge on *J. glutinosa*, focusing on its traditional uses, phytochemical composition, and pharmacological activity. Particular emphasis is placed on pharmacodynamics and ethnopharmacology, specifically the relationship between its pharmacological properties, its possible mechanism of action, and its uses in folk medicine.

Understanding the mechanism of action of a substance in relation to its chemical structure provides crucial insights into its efficacy, potential adverse effects, and possible interactions.

Information regarding the traditional uses, phytochemical composition, pharmacological activity, and mechanisms of action of *J. glutinosa* was obtained through a literature search across several scientific databases, including Web of Science, PubMed, Google Scholar, and Wiley Online Library. The primary search employed the keywords “*Jasonia glutinosa* or *Chiliadenus*”, followed by additional terms such as “pharmacological activity”, “phytochemistry”, or “traditional uses”.

Studies lacking sufficient data on the chemical or pharmacological composition were excluded. Given the limited literature available on this plant, no restrictions were imposed based on publication year, and the search covered all relevant studies up to 2024. Articles were selected by reviewing titles and abstracts, ensuring the inclusion of only relevant studies.

## 2. Ethnomedicinal/Traditional Uses

The exact time when the infusion of this plant first began to be consumed remains unknown. However, it was not until 1867 that one of the common names of *Jasonia glutinosa*, “té de Aragón,” was documented. Today, *J. glutinosa* is considered one of the most popular medicinal plants in Spain and is widely used in almost all the regions where it grows [2].

As previously mentioned, *J. glutinosa* is primarily used in the form of an infusion for gastrointestinal disorders. However, depending on the region of Spain, it has additional medicinal applications and preparation methods (see Table 1). This explains why most of its common names derive from the word “té” and refer to its consumption as a tea-like infusion, typically for digestive purposes [1,4,5].

Beyond its use as an infusion, it is also utilized in the production of herbal liqueurs, which are consumed as digestives. In certain regions, it is used to treat wounds, bumps, and bruises, earning it the common name “arnica” [1]. Additionally, its venotonic properties have led to its designation as the “blood herb” due to its use in lowering blood pressure and treating anemia [1].

As observed, the therapeutic properties of *J. glutinosa* documented in the literature are closely associated with its common names. No records of its medicinal use have been found in the other regions where it grows.

Apart from medicinal use in humans, *J. glutinosa* has also been used in animals. Its main veterinary use has been in ruminants as an infusion to treat bloating, a digestive disorder caused by excessive gas accumulation. It is also used topically to wash and heal wounds in animals and to treat mouth sores in horses [2].

## 3. Phytochemical Composition

Numerous phytochemical studies of *J. glutinosa* have been conducted on different parts of the plant using various types of extracts. Both volatile and non-volatile compounds have been identified through diverse methods and techniques.

The volatile compounds of *J. glutinosa* essential oil were first studied by Guillén and Ibargoitia, who obtained both the essential oil and a pentane extract from the dried leaves [10]. This study revealed that both extracts, the essential oils and the pentane extract, contained a high number of oxygen monoterpene and sesquiterpene derivatives. The main compounds were the monoterpenes camphor (42.4 and 38.3%, respectively, in essential oil and pentane extract) and, to a lesser extent, endo-borneol. Other volatile constituents identified were the monoterpene α-terpineol and the sesquiterpenes nerolidol and T-cadinol [10].

González Romero et al. used the distillation from the fresh leaves of the plant to obtain the essential oil [11]. Their analysis also identified the monoterpenes camphor and borneol as the main volatile compounds in the essential oil. Other terpenes identified were the sesquiterpenes caryophyllene oxide, farnesol, cadinol, and spatulenol, as well as the monoterpene bornyl formate, along with α-pinene, eucalyptol, and linalool [11].

Pascual Teresa et al. identified a novel sesquiterpene alcohol, kudtdiol, from a benzene extract of the aerial parts of *J. glutinosa*. The major sesquiterpenic component was characterized as (−)-[11R]-4α-hydroxy-eudesm-11,12-diol [12]. Subsequently, three new eudesmane alcohols were also reported by the same authors, using spectroscopic methods and chemical correlations. These compounds were α-epoxy kudtdiol ((−)-[11R]-4α,14-epoxyeudesm-11,12-diol), 5-epi-kudtriol ((−)-[11R]-eudesm-4(14)-en-5β,11,12-triol), and kudtriol ((+)-[11R]-eudesm-4(14)-en-5α,11,12-triol) [13].

Villaescusa Castillo et al. identified two new sesquiterpenoids from a MeOH-CH_2_C_12_ extract obtained from the dried aerial parts of *J. glutinosa*. These compounds were lucinone (β5, 11,12-trihydroxy-iphionan-4-one) and glutinone (2-[5′-(2′-oxopentyl)]-2-methyl-5-(l’- hydroxy- l’-methylethanol)-cyclohexanone) [14]. Furthermore, two new eudesmane alcohols from these species were isolated and identified from acetone/water extract of aerial parts of *J. glutinosa*. These compounds were the (11 R)-eudesm-4-en-l 1,12-diol and (11 R)-eudesmane-5α,l 1,12-triol [15].

On the other hand, the non-volatile compounds of the plant have been extracted by different methods, generally, through maceration with different solvents. The research group of Villaescusa Castillo et al. also identified a high total polyphenol content from the hydroalcoholic extracts of the aerial part in *J. glutinosa*. From the MeOH/H_2_O extract, five methyl flavonol glucopyranosides were identified: patuletin-7-O-β-D-glucopyranoside, patuletin-3-O-β-D-glucopyranoside, quercetin-3-O-β-D-glucopyranoside, quercetin-3-O-β-D-galactopyranoside and quercetin-7-O-monoglucoside [16]. From the n-BuOH extract of the aerial part, the elucidated flavonol glucuronosides included kaempferol-3-O-β-D-glucuronopyranoside, quercetin-3-O-β-D-glucuronopyranoside, and quercetin-3-O-β-D-glucuronopyranoside-6″-methyl ester [17].

Ortega-Vidal et al. conducted extractions using MeOH and H_2_O from five commercial rock tea samples. Although the aqueous extract presented higher amounts of phenolic content compared to the MeOH one, both extracts were particularly rich in caffeoylquinic acids. Other compounds detected were phenolic acids such as citric acid and benzoic acid and flavonoids such as mearnsetin glycosides, quercetin, vicenin-2, rutin, kaempferol, isorhamnetin, and other glycoside derivatives. This work also simulated the chemical digestion of the extracts and observed that although a large amount of the compound was lost during this process, various dicaffeoylquinic acids remained intact [18].

Valero et al. analyzed an ethanolic extract of *J. glutinosa* by HPLC-MS, finding an extract rich in flavonoids, namely patuletin glucopyranoside as the main compound [19]. Further analysis by HPLC–DAD revealed that the extract contained a substantial number of phenolic compounds and pigments. A total of 15 phenolic compounds were identified, including 10 phenolic acids and five flavones. The 70% of the total phenolic content was represented by 3,4-di-O-caffeoylquinic acid, 3,5-di-O-caffeoylquinic acid, 4,5-di-O-caffeoylquinic acid, and 1,5-di-O-caffeoylquinic acid. The flavonoid quercetin-3-O-galactoside represented 50% of the total flavonoid content. Regarding pigments, lutein represented 55% of the total, along with two carotenoids, chlorophylls, and xanthophylls [20].

Mohammed et al. performed a phytochemical study of a 70% methanolic extract of *J. glutinosa* collected in Libya [21]. Their analyses revealed the presence of abundant phenolic acids and flavonoid derivatives, particularly cinnamic acid derivatives, including caffeoylquinic and dicaffeoylquinic glycosides. Other flavonoids identified included quercetin and kaempferol glycosides, isorhamnetin, and the isoflavone 3′-O-methylorobol, as well as pigments such as lutein.

In summary, extracts rich in volatile compounds, such as essential oils, are rich in secondary metabolites from the terpene group, notably camphor, borneol, and eudesmane alcohols. Conversely, non-volatile compounds are primarily composed of polyphenols, which represent the main group of secondary metabolites and are predominantly found in hydroalcoholic extracts. These include flavonol glycosides such as quercetin, patuletin, or kaempferol; phenolic acids such as caffeoylquinic acids; and pigments such as lutein, carotenoids, chlorophylls, and xanthophylls (Table 2). The diversity in the phytochemical composition is attributed to the different extraction methods used, the various plant parts analyzed, and the inherent variability of the plant, depending on the season and the geographic location of collection.

## 4. Pharmacological Activities and Possible Molecular Mechanisms of Action

**Figure 2:**
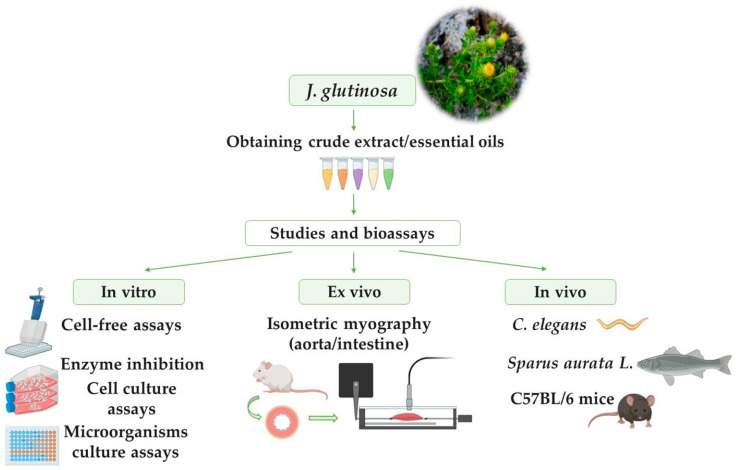
Different methodologies, including in vitro, ex vivo, and in vivo, carried out with *J. glutinosa* to study its biological activities.

Recent in vitro, ex vivo, and in vivo studies have demonstrated various pharmacological properties of *J. glutinosa*, including antioxidant, anti-inflammatory, antimicrobial, and other biological activities [18,19,20,21,22,23,24,25,26,27,28] (Figure 2). These pharmacological effects are attributed to the plant’s phytochemical composition. However, only a limited number of studies have isolated specific compounds from its extracts and evaluated their bioactivity. Table 3 summarizes the pharmacological activities, study types, mechanisms of action, and the compounds potentially responsible.

### 4.1. In Vitro Studies

#### 4.1.1. Antimicrobial Activity

Villaescusa-Castillo et al. evaluated the effect of acetone extract from *J. glutinosa* against *Entamoeba histolytica*, *Leishmania donovani*, and *Trichomonas vaginalis*. The extract showed leishmanicidal activity at 100 µg/mL and amoebicidal activity at 250 µg/mL, but it exhibited no trichomonacidal activity. The antiparasitic activity of two sesquiterpenes alcohols, 5-epi-kutdtriol and kutdtriol, isolated from the acetone extract, was studied by Villaescusa et al. To do this, they tested the effect of these compounds against *Leishmania donovani* and *Plasmodium falciparum.* Kutdtriol, but not 5-epi-kudtriol, showed leishmanicidal and antimalarial activity at 250 µg/mL [23].

López et al. studied the antifungal activity of ethyl acetate (EtOAc), methanol (MeOH), aqueous (H_2_O), and dichloromethane (DCM) extracts obtained from the aerial parts of *J*. *glutinosa*. They used the fungus *Rhizopus stolonifer* and evaluated the antifungal activity using the mycelial growth method. Of the four extracts tested, only the DMC extract inhibited filamentous fungi (MIC > 1000 μg/mL). This antimicrobial activity is likely associated with the presence of sesquiterpenoids [24].

Although Mohammed et al. recently reported the absence of antibacterial activity in the ethanolic extract of *J. glutinosa* [21], several studies have documented antibacterial properties in some of its main constituents [29,30,31].

Sesquiterpenoids possess significant potential as natural antimicrobial agents due to their lipophilic nature and low polarity, which facilitate penetration through the cell wall, leading to structural disruption. For instance, artemisinin, a sesquiterpene lactone, is widely used as an antimalarial agent [32,33]. Several sesquiterpenes found in *J. glutinosa* have demonstrated antimicrobial activity. Farnesol, identified in *J. glutinosa*, has been reported to disrupt microbial cell membranes [34]. Furthermore, it can degrade biofilms of Gram-positive bacteria by reducing their biomass. This activity may be partly attributed to its hydrophobic nature, which would facilitate its insertion into the bacterial phospholipids bilayer and cause structural alteration. Similarly, β-caryophyllene, a compound found in *J. glutinosa*, damages the cell membrane and produces non-selective pore formation, leading to the leakage of cellular contents and finally resulting in cell death [32,33]. Likewise, the flavonoids, caffeoylquinic acid, and pigments present in this species have also shown antimicrobial and antifungal properties [30,35,36].

#### 4.1.2. Antioxidant Activity

Several studies have investigated the antioxidant effect of *J. glutinosa*. López et al. also evaluated the radical scavenging capacity of EtOAc, MeOH, H_2_O, and DCM extracts from *J. glutinosa,* using the 2,2-diphenyl-1-picrylhydrazyl (DPPH) free radical method, by rapid TLC screening and a spectrophotometric assay. DPPH is stable at room temperature and produces a violet solution in methanol. In the presence of an antioxidant molecule capable of donating hydrogen, it is reduced, resulting in a colorless solutions [24]. All extracts presented antioxidant properties, with the MeOH and H_2_O extracts showing the highest activity (IC_50_ > 2000 µg/mL for DCM, 283.71 ± 22.5 µg/mL for EtOAc, 31.32 ± 1.5 µg/mL for H_2_O, and 29.8 ± 3.9 µg/mL for MeOH) [24].

In the same way, Ortega-Vidal et al. assessed the antioxidant activity of a methanolic and aqueous extracts of *J. glutinosa* using the DPPH and ABTS radical scavenging assays. Both extracts presented antioxidant activity, with the ABTS values being higher for the aqueous extract (17.9 vs. 15.3 g trolox/100 g of dried extracts) and the DPPH values being higher for the methanolic extract (17.4 vs. 7.0 g trolox/100 g of dried extracts). After simulated chemical digestion to mimic intestinal processing, both extracts showed a reduction in antioxidant capacity, likely due to the degradation of phenolic compounds during digestion [18]. Espinosa et al. also studied the antioxidant properties of *J. glutinosa* assessed by the DPPH assay. The ethanolic extract of *J. glutinosa* demonstrated greater free radical scavenging capacity at low doses compared to the aqueous extract. However, at high doses (1 and 5 mg/mL), both extracts showed similar DPPH inhibition capacities. The same results were observed for the reducing power test [25].

Valero et al. demonstrated antioxidant activity in cell-free assays. First, the antiradical properties were studied using a non-enzymatic system (NADH/PMS system) and then by an enzymatic system involving the xanthine/xanthine oxidase (X/XO) system. *J. glutinosa* extract, in a dose-dependent manner, eliminated the formation of superoxide radicals generated in both systems (IC_50_ of 47.0 μg/mL IC_50_ of 36.7 μg/mL, respectively). Furthermore, the effect of *J. glutinosa* on XO activity was studied by measuring the formation of uric acid from xanthine. The extract showed the ability to inhibit the XO activity (IC_50_ value of 929.86 μg/mL), demonstrating that it can scavenge superoxide radicals through different pathways [20]. Other authors have also demonstrated the antiradical activity of *J. glutinosa* extract using the oxygen radical antioxidant capacity (ORAC) assay, with a value of 2.72 μmol TE per mg of extract, and ferric reducing antioxidant power (FRAP) assays, with a value of 59.09 μmol Fe^2+^ per g of extract [21,26].

The antioxidant activity of compounds isolated from *J. glutinosa* has not been specifically studied. However, this pharmacological property is attributed to the plant’s content of phenolic compounds, since the compound–activity correlation is strong. The mechanisms through which these compounds exert their antioxidant action are partly due to their ability to chelate transition metals (Fe^2+^, Cu^2+^, Zn^2+^) and scavenge free radicals. Additionally, they inhibit oxidases such as lipoxygenase (LOX), cyclooxygenase (COX), myeloperoxidase (MPO), NADPH oxidase, and XO, preventing the generation of reactive oxygen species (ROS) and organic hydroperoxides in the body. They also act on other enzymes, such as phospholipase A2, while stimulating antioxidant enzymes such as catalase (CAT) and superoxide dismutase (SOD) [37].

Caffeoylquinic acids, present in the ethanolic extract of *J. glutinosa*, have exhibited antioxidant activity similar to that of vitamin E [38]. Furthermore, these compounds have been shown to prevent protein oxidation, lipid peroxidation, and hydroxyl radical formation [38].

#### 4.1.3. Anti-Inflammatory Activity

The anti-inflammatory capacity of ethanolic *J. glutinosa* extract was studied in murine macrophages (J774.2). In this study, macrophages were stimulated with LPS, and the production of nitric oxide (NO) and the expression of the pro-inflammatory cytokine tumor necrosis factor alpha (TNF-α) were measured. *J. glutinosa* extract, at a dose of 94 μg/mL, decreased both NO production and TNF-α expression in LPS-stimulated macrophages, bringing them back to basal levels [20]. Additionally, in a cell-free experiment, the extract decreased the activity of 5-lipoxygenase (5-LOX), a necessary enzyme for leukotriene synthesis, with an IC_50_ of 74.0 μg/mL [20]. Leukotrienes are constrictors in smooth muscles, play a role in inflammatory processes, and increase vascular permeability. Therefore, 5-LOX inhibitors are considered an important therapeutic target for the treatment of coronary artery disease or asthma [39].

Espinosa et al. studied the innate cellular immunity in leukocytes obtained from head and kidney samples (HK leukocytes) of fish fed diets containing 10% or 30% *J. glutinosa,* and the results were compared with those obtained by the control group (fish fed without supplementation) [25]. Flow cytometry was used to study the phagocytic ability of HK leukocytes to phagocytose *Saccharomyces cerevisiae* (strain S288C). The phagocytic ability of fish supplemented with both doses of *J. glutinosa* for 15 days was significantly higher than that of the control group. However, no differences were observed after 30 days. The authors concluded that *J. glutinosa* has an immunostimulant effect after 15 days of administration, and attributed the absence of this effect after 4 weeks to the adaptation of the immune system to the new diet. Regarding respiratory burst activity, only the fish fed the 10% *J. glutinosa* diet for 30 days showed a significantly higher response in HK leukocytes compared to the control group. No changes in peroxidase activity in HK leukocites were observed in any of the groups [25].

Four sesquiterpenes isolated from *J. glutinosa*, lucinone (100 µM), glutinone (25 µM), 5-epikutdtriol (50 µM), and kutdtriol (100 µM), have demonstrated anti-inflammatory activity in cellular systems that generate metabolites of cyclooxygenase 1 (COX-1) and 5-LOX [27]. Arachidonic acid is the precursor to various eicosanoids, such as prostanoids (thromboxanes and prostaglandins) obtained by COX; leukotrienes and lipoxins, generated by LOX; and hydroeicosatetraenoic acids and epoxyeicosatrienoic acids, generated by cytochrome P450. Eicosanoids exert a crucial role in mediating inflammatory response [39,40].

The stimulation of mouse peritoneal cells by the calcium ionophore A23187 resulted in the release of prostaglandin E_2_ (PGE_2_) and leukotriene C_4_ (LTC_4_). Treatment of these cells with the sesquiterpenes showed a significant reduction in the release of PGE_2_, but not LTC_4_, compared to control groups (cells incubated with reference compounds). The percentage of inhibition of PGE-release was as follows: lucinone (61.97%), glutinone (70.59%), 5-epikutdtriol (65.88%), and kutdtriol (76.67%). All the sesquiterpenes exhibited the capacity to inhibit COX-1, but not 5-LOX, with the inhibition being most pronounced with kutdtriol (IC_50_ values of 39 µM) [27]. Although the results indicated that none of the sesquiterpenes tested inhibited the 5-LOX enzyme, Valero et al. demonstrated that the *J. glutinosa* extract itself inhibited the 5-LOX enzyme [20]. In human platelets, only glutinone showed a significant inhibitory effect on thromboxane B2 (TBX_2_) release (IC_50_ values of 24 µM) [27]. Therefore, this suggests that another compound in the extract is responsible for this activity.

Flavonoids such as quercetin and kaempferol, caffeoylquinic acids, and carotenoids such as lutein, found in the *J.glutinosa,* present anti-inflammatory activity due to their ability to inhibit inflammatory enzymes such as COX, LOX, and iNOS, as well as other pro-inflammatory mediators such as NO and cytokines (IL1β, IL-6, IL-8, TNF-α). They also suppress the activation of the transcription factor nuclear factor kappa β (NF-κβ) [29,35,36,41]. In addition, flavonoids, such as those found in propolis, are used to combat inflammatory processes due to their immunomodulatory activity [30].

#### 4.1.4. Neuroprotective Properties

The ability of the ethanolic extract of *J. glutinosa* to inhibit enzymes of the nervous system, such as acetylcholinesterase (AChE), monoamine oxidase (MAO), and tyrosinase (TYR), was studied. These enzymes participate in the metabolism of neurotransmitters and play a role in the development of different neurological disorders, such as Alzheimer’s or Parkinson’s diseases. The extract showed the ability to inhibit the three central nervous system (CNS) enzymes, with a IC_50_ values of 4.5 mg/mL, 76.34 µg/mL, and 5 mg/mL, respectively [28]. Our research group has also studied other compounds isolated from plant extracts and flavonoids, which have demonstrated the ability to inhibit CNS enzymes [42,43,44].

#### 4.1.5. Antihypertensive Effects

Whole-cell clamp experiments were performed using the rat aortic smooth muscle cell line A7r5, which expresses the L-type Ca^2+^ channel. Incubation with ethanolic *J. glutinosa* extract (2.5 mg/mL) resulted in the blocking of these calcium channels [19]. The mechanism of action and the molecules potentially responsible for this effect will be discussed in a later section.

#### 4.1.6. Anti-Obesity and Antidiabetic Activity

Using enzyme inhibition assays, Les, et al. studied the ability of the ethanolic *J. glutinosa* extract to inhibit the activity of digestive enzymes. The extract demonstrated the capacity to inhibit α-glucosidase (α-GLU), a brush border enzyme that hydrolyzes starch and disaccharides to release glucose for intestinal absorption, and lipase, an enzyme that hydrolyze ester bonds in lipids and releases free fatty acids, in a dose-dependent manner (IC_50_ values of 2052.32 ± 895.14 µg/mL and 891.50 ± 65.4 µg/mL, respectively). Only at high doses, the extract also inhibited fatty acid amide hydrolase (FAAH) (IC_50_ values of 118.39 ± 98.11 µg/mL) [26], an enzyme that hydrolyzes fatty acid amides, thereby regulating the levels and duration of gene expression mediated by lipid mediator, such as palmitoylethanolamide and oleoylethanolamide [45]. On the other hand, murine 3T3-L1 preadipocytes were used to study the anti-obesity and antidiabetic activities of this extract. 3T3-L1 adipocyte-like cells treated with differentiation medium developed into enlarged adipocytes. However, supplementation of the medium with various concentrations of extract doses (0, 10, 50, and 100 μg/mL) reduced, in a dose-dependent manner, the number of differentiated preadipocytes and mature adipocytes. Similarly, the amount of fat in the adipocytes was studied using oil red O staining, which revealed that cells treated with the extract contained fewer lipid droplets inside. This inhibitory effect on adipogenesis was confirmed by the quantification of the lipophilic dye [26].

In addition, these authors investigated whether *J. glutinosa* extract has a lipid-reducing effect. To do this, they quantified the triglyceride (TG) content in the cells. The results showed that the extract significantly decreased the accumulation of TG in adipocytes [26].

All these results demonstrate that *J. glutinosa* extract reduces fat accumulation during adipocyte maturation and TG accumulation in the mature adipocyte, revealing, for the first time, its anti-adipogenic activity and delipidating effects.

*J. glutinosa* could exert its activities due to its high content of caffeoylquinic acids, which modulate glucose metabolism. Caffeoylquinic acids are a type of insulin sensitizer, enhancing its action and preventing postprandial hyperglycemia by delaying glucose absorption in the small intestine through the inhibition of glucose-6-phosphate translocase 1. Additionally, they inhibit digestive enzymes such as α-amylase, β-GLU, and lipase. Furthermore, these compounds decrease the expression of hepatic glucose-6-phosphatase, affecting glycogenolysis and gluconeogenesis, and stimulate the activation of AMPK in the skeletal muscle, promoting glucose uptake in this tissue. They also increase the secretion of adiponectin and its receptors. All these effects contribute to lower levels of TG, cholesterol, glycated hemoglobin, and fasting glucose, as well as increased glucose tolerance and insulin sensitivity [35,38]. Moreover, caffeoylquinic acids inhibit aldose reductase, preventing the abnormal accumulation of intracellular sorbitol in diabetic patients, and inhibit protein tyrosine phosphatase 1B, a target involved in the treatment of diabetes and obesity [29].

### 4.2. Ex Vivo Studies

#### 4.2.1. Antihypertensive Effect

Ethanolic *J. glutinosa* extract has demonstrated a dose-dependent vasorelaxant effect in rat aortic rings pre-contracted with KCl, a depolarizing agent, and phenylephrine, an α_1_-adrenoceptor agonist, by isometric myography [19]. The vasorelaxant effect produced by the extract in KCl-precontracted rings was similar to that induced by verapamil, a blocker of L-type Ca^2+^ channels (EC_50_ value of 2.4 mg/mL vs. 0.32 μM, respectively).

This study suggests that the antihypertensive action of *J. glutinosa* may involve the blockade of Ca^2+^ entry from the extracellular medium. In a calcium-free medium, *J. glutinosa* (5 mg/mL) shifted the muscle contraction curve produced by ClCa_2_ downwards and rightwards, in a manner similar to verapamil (10^−6^ M). Furthermore, pre-incubation of the aortic rings with the extract blocked the effect of BayK 8644, an L-type Ca^2+^-channel agonist (93 ± 1% vs. 97 ± 2%, respectively). It is important to remember that this inhibition of the calcium channel was observed in vitro. These authors also suggest that *J. glutinosa* extract could inhibit calcium efflux from the sarcoplasmic reticulum and modulate the RhoA/Rho-kinase signaling pathway [19].

The authors speculated that this inhibition could be caused by flavonoids and terpenes, which, in addition to their antioxidant activity, may enhance cardiovascular function by blocking Ca^2+^ channels [46]. Flavonoid-rich foods, such as *J. glutinosa*, have been associated with improvement in endothelial function and a decrease in hypertension and cardiovascular disease. These effects are likely due to the vasorelaxant activity, which may occur through various pathways affecting the endothelial cells and/or the vascular smooth muscle [46].

On the other hand, caffeoylquinic acids, abundant in the ethanolic extract of *J. glutinosa*, could also contribute to hypotensive activity. These acids have been shown to influence NO levels, producing a vasodilatory effect in rat vessels. In clinical studies, they have reduced both systolic and diastolic blood pressure in hypertensive patients, without any adverse effects [35]. Moreover, their antioxidant and anti-lipidemic properties help prevent the development and progression of atherosclerotic disease and coronary heart disease [29]. Lutein, another compound found in *J. glutinosa*, demonstrated beneficial effects in preventing and managing atherosclerosis and its risk factors, such as inflammation and endothelial dysfunction. Lutein has been shown to reduce blood pressure, arterial thickness, and the migration of monocytes and vascular smooth muscle cells [47].

#### 4.2.2. Antispasmodic Activity

In this study, the isometric motility of rat (Wistar, male) duodenal segments was measured using an organ bath [22]. The extract of *J. glutinosa* induced a relaxant effect on spontaneous contractions, with EC_50_ values of 2.29 mg/mL. In addition, the extract significantly reduced, in a dose-dependent manner, the amplitude of spontaneous contractions, although it did not affect the frequency. Moreover, the extract relaxed the duodenal smooth muscle pre-contracted with KCl, CaCl_2_, and Bay K8644, an L-type Ca^2+^ channel agonist. Verapamil (10^−6^ M) induced an inhibition similar to that produced by 5 mg/mL of the extract. These findings demonstrated that *J. glutinosa* possesses spasmolytic activity mediated by the inhibition of L-type Ca^2+^ channels.

Another study analyzed the ileal motility of healthy and colitic mice using isometric myography [20]. The animals were divided into different treatment groups: water or *J. glutinosa* extract (5, 25 or 50 mg/kg body weight) by oral gavage for 20 days. The animals were further categorized as “healthy” if they received drinking water or “colitic” if they received dextran sulfate sodium (DSS, 2.5%) in drinking water starting on day 11. After completing the assay, the ileums were collected and placed in an organ bath. *J. glutinosa* extract itself did not modify the typical contractile activity of the longitudinal smooth muscle of the ileum. DSS produced dysmotility, significantly decreasing the amplitude and, to a lesser extent, the frequency of spontaneous contractions of the ileum. Treatment with the extract reversed the DSS effect in a dose-dependent manner. The highest dose of *J. glutinosa* extract (50 mg/kg) restored coordinated rhythmic spontaneous motility, similar to that of control animals [20]. Furthermore, the study evaluated the ileum’s contraction capacity in response to acetylcholinesterase (ACh, 10^−4^ M). Colitic animals showed significantly decreased contractions produced by ACh, but treatment with the extract at higher doses prevented this effect [20]. The study concludes that treatment with *J. glutinosa*, in a dose-dependent manner, improved ileum functionality, including both motility and contractile capacity, in mice with ulcerative colitis.

As discussed above, flavones and terpenes have been shown to block calcium channels in smooth muscle, including both vascular and intestinal smooth muscle [48,49].

### 4.3. In Vivo Studies

#### 4.3.1. Antioxidant Activity

Espinosa et al. investigated the antioxidant potential of *J. glutinosa* as a dietary supplement for gilthead seabream (*Sparus aurata* L.). The fish were fed experimental diets containing 0% (control), 10%, or 30% *J. glutinosa* for 15 and 30 days. At the end of the treatment, different biomarkers related to stress/oxidative stress, such as protein oxidation and Heat Shock Protein 70 (HSP 70), were analyzed in skin mucus [25].

The level of protein oxidation was significantly lower after 30 days in the mucus of fish supplemented with 30% *J. glutinosa* compared to the control group. The oxidation of amino acids leads to the formation of carbonyl groups in proteins, and the increase in these groups has been related to aging and various human pathologies. Another biomarker studied was HSP 70, a chaperone involved in stabilizing protein folding, which is crucial for maintaining correct protein structure and function. Through immunoblotting of skin mucus, they observed that after 15 days, the animals supplemented with *J. glutinosa* showed slightly lower levels of HSP70 compared to the control group. After 30 days, only fish fed with 30% *J. glutinosa* showed significantly lower HPS 70 levels than the control group. Similarly, the expression of the HSP 70 gene in the liver of animals supplemented with *J. glutinosa* after 30 days was significantly reduced compared to the control group [25].

In addition, the expression of two antioxidant enzymes, CAT and SOD, as well as a stress-related gene, NFE2L2, which encodes nuclear factor erythroid 2 (Nrf2), a transcription factor important for regulating the expression of antioxidant response genes, was studied in the liver and gut of gilthead seabream. The expression of CAT, SOD, and Nrf2 was increased after 15 days in both the liver and gut. However, at 30 days, these changes were only maintained in the expression of SOD in the fish supplemented with 30% *J. glutinosa*. This effect may explain the decrease in carbonyl groups observed in skin mucus sampled after 30 days [25].

Once again, the authors suggest that the high content of total polyphenols in *J. glutinosa* extracts, particularly polyphenols and flavonoids, are responsible for its antioxidant activity. These compounds are thought to enhance protection against oxidative stress by reducing HSP 70 levels, increasing the activity of antioxidant enzymes such as CAT and SOD through Nrf2 signaling, and preventing protein oxidation.

Les et al. studied the antioxidant capacity of *J. glutinosa* using the *Caenorhabditis elegans* (*C. elegans*) model, assessing the survival rate percentage through the Lethal Oxidative Stress Resistance Test and Lifespan Analysis. Wild-type nematodes were pretreated with different doses of ethanolic extract of *J. glutinosa* for 24 h, followed by the addition of juglone, a natural pro-oxidant, to the medium. The *J. glutinosa* extract improved the survival rate of nematodes in a dose-dependent manner, with a 10.69% increase in survival rate at 50 mg/mL, showing a protective effect against oxidative stress [28]. On the other hand, as oxidative stress is related to aging, the authors also evaluated the nematodes’ lifespan curves to study the extract’s antioxidant capacity. The results demonstrated that *J. glutinosa* increased the lifespan of the worms in a dose-dependent manner, with a 13.81% increase at 100 mg/mL [28]. The antioxidant properties and lifespan extension produced by *J. glutinosa* extract were similar to those of two flavonoids present in the extract, quercetin and kaempferol [50].

The supplementation in the diet with *J. glutinosa* could enhance resistance to stress and provide an antioxidant effect.

#### 4.3.2. Anti-Inflammatory Activity

The administration of DSS produces damage and inflammation mainly in the colon and other parts of the intestine. This inflammation is observed at the microscopic level, with massive destruction of the intestinal mucosa and severe infiltration of inflammatory cells into the *lamina propria*. Anatomically, there is an increase in colon wall thickness and a reduction in its size. Treatment with *J. glutinosa* reversed the effects of DSS in a dose-dependent manner [20]. The histological study of the colon of colitic animals treated with *J. glutinosa* (50 mg/kg) showed a typical colon structure, with intact intestinal *villi* and a normal *lamina propria*, presenting a scant cellular infiltrate in the submucosa. The length and wall thickness of the colon in these animals were similar to those of the control animals. The efficacy of *J. glutinosa* was similar to that of sulfasalazine (SSZ, 100 mg/kg), a standard anti-inflammatory used in the treatment of ulcerative colitis [20]. Once the experimental protocol was finished, various inflammatory biomarkers in the colonic tissue were analyzed. Treatment with *J. glutinosa* decreased the MPO activity, IL-6 levels, and the protein expression of iNOS and COX-2, compared to untreated colitic animals [20]. The administration of the *J. glutinosa* extract alone in the healthy animals did not cause any alteration in the tissue structure or inflammatory biomarkers, suggesting that the extract’s dose was not toxic [20].

In the study by Espinosa et al. humoral immune parameters were also studied in the serum and skin mucous from gilthead seabream, including peroxidase activity, antibodies (IgM), complement factors, and other lytic factors, which act as a first line of defense, preventing microbial adhesion and colonization. An increase in peroxidase activity was observed in the skin mucus of fish supplemented with 10% *J. glutinosa* for 15 days, but not for 30 days, compared to the control group. The authors suggest that this effect could be due to the animals adapting to the diet. Regarding the IgM levels in skin mucus, no significant differences were observed between the different groups. However, serum from fish supplemented with 30% *J. glutinosa* for 30 days showed significantly higher IgM levels than the control group. The analysis of complement activity in serum did not show any significant differences between the study groups [25].

Furthermore, the expression of four genes related to the immune system (IL1β, TNF-α, βDEF, and MHCII) was studied in the liver and proximal gut of gilthead seabream [25]. No differences were observed in the expression of TNFα, βDEF, and MHCII between the different groups. Interestingly, IL1β gene expression in the liver was significantly increased in fish fed *J. glutinosa* for 15 days, but decreased at 30 days, compared to the control diet. IL1β is a potent pro-inflammatory cytokine produced by activated macrophages and acts as an important mediator in immune and inflammatory response. These results are consistent with these authors’ findings regarding phagocytic capacity and respiratory status. The changes in IL1β expression further suggest that *J. glutinosa* in the diet may have immunostimulant properties. No significant differences in the expression of these genes were found in gut samples across different groups [25].

As mentioned above, terpenes isolated from *J. glutinosa* have demonstrated anti-inflammatory capacity in vitro. In addition, other compounds found in the extract, such as quercetin, isorhamnetin, kaempferol, and caffeoylquinic acids, have demonstrated anti-inflammatory activity in in vivo models [35,51].

#### 4.3.3. Digestive Activity

*J. glutinosa* has been widely used for its digestive properties in the treatment of gastrointestinal disorders. However, scientific studies investigating its effects on the digestive system and underlying mechanisms of action remain limited. The following section highlights two pharmacological activities of this species related to its digestive properties.

##### Antispasmodic Activity

In a murine model of colitis induced by the administration of DSS in the drinking water to male C57BL/6 mice, gastrointestinal transit was measured [22]. To assess gastrointestinal transit, 200 µL of a solution containing 5% Arabic gum and non-absorbable Evans blue dye was administered via intragastric gavage. Two groups were formed: group 1 received ethanolic *J. glutinosa* extract (50 mg/kg), and group 2 received water (vehicle) for 17 days. DSS (2.5%) was administered from day 10 to day 17 to induce colitis. Gastrointestinal transit was measured on day 1 (basal control) and day 7, to study the effect of the extract, and day 17, to evaluate the extract’s protective effect in the murine colitis model. The *J. glutinosa* extract and the vehicle did not alter gastrointestinal transit time in healthy animals. Nevertheless, DSS treatment accelerated transit time in vehicle-treated animals; however, the extract reverted this increases in gastrointestinal transit time [22].

##### Improvement of Inflammatory Bowel Disease

Inflammatory bowel disease, such as ulcerative colitis or Chron’s disease, are pathologies whose incidence and prevalence have been increasing over the years. The symptoms of these diseases are often similar, including weight loss, the presence of blood in the stool, abdominal pain, and alterations in intestinal motility [52]. Conventional treatments for these pathologies are often not fully satisfactory, leading patients to search alternative therapies, such as phytotherapy [53].

In a lesion–repair colitis model, ethanolic *J. glutinosa* extract was shown to have a protective effect, in a dose-dependent manner, on the development of DSS-induced colitis in mice, and it improved their recovery. These pathologies are characterized by damage to the intestinal mucosa, increased permeability of the intestinal barrier, and an inflammatory response, all of which disrupt gastrointestinal motility. Maintaining the integrity of the intestinal barrier is essential for proper intestinal function. Tight junction proteins between epithelial cells play a key role in maintaining the intestinal barrier. This study demonstrated that the extract preserves the expression of the tight junction protein ZO-1 in enterocytes [20]. This would explain why the animals treated with *J. glutinosa* presented a lower inflammatory response, less tissue damage, and, therefore, a delay in the onset of typical colitis symptoms (weight loss, the presence of blood in the feces, and changes in the consistency of feces) and recovered more quickly once DSS was removed compared to untreated animals or animals treated with the reference drug SSZ [20].

The protective effect of *J. glutinosa* in a murine model of colitis and its mechanism of action have been similar to those observed in in vivo models with other compounds found in the extract, such as quercetin, kaempferol, and other flavonoids [41].

#### 4.3.4. Neuroprotective Properties

The neuroprotective capacity of the ethanolic extract of *J. glutinosa* was evaluated in the *C. elegans* model [28]. For this purpose, the transgenic strain CL4176 of *C. elegans* was used as a model of Alzheimer’s disease. This strain, when exposed to increased incubation temperatures, induces the expression of the human amyloid Aβ transgene, which produces progressive paralysis in the worm. The extract, independent of dose, decreased Aβ-induced paralysis in transgenic *C. elegans* by 21–28%. The time at which 50% of the worms became paralyzed was significantly increased with *J. glutinosa* supplementation compared to control groups. *J. glutinosa* showed neuroprotective capacity in worms, preventing the risk of paralysis [28].

It was suggested that the caffeoylquinic acids in the extract were responsible for this neuroprotective activity. As previously mentioned, these compounds have strong antioxidant and anti-inflammatory properties, which likely contribute to their neuroprotective capacity. They have shown neuroprotective effects, reducing toxicity induced by the amyloid Aβ peptide, preventing cell death in the nervous system and exhibiting neurotrophic effects similar to those of nerve growth factor [29].

## 5. Current Situation and Perspectives

**Figure 3:**
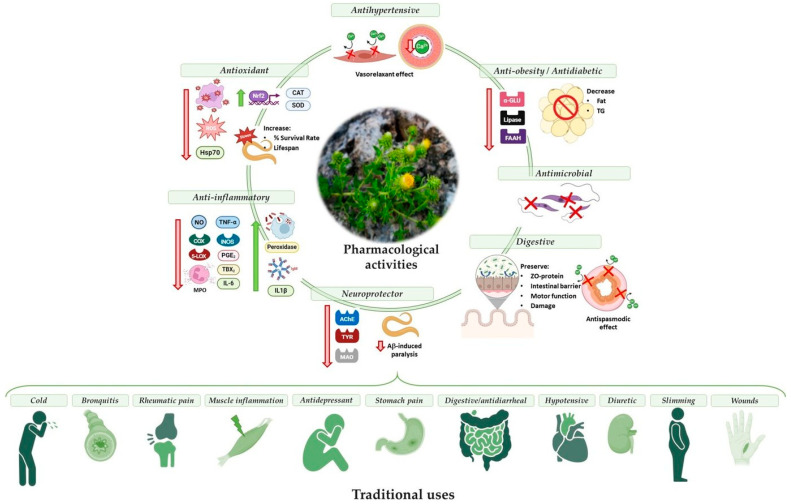
Pharmacological activities and molecular action mechanisms that may explain the traditional ethnopharmacological use of *Jasonia glutinosa*.

This plant species has been used as an elixir or universal remedy for a wide range of disorders based on traditional and cultural practices. However, the studies presented on the different pharmacological properties of *J. glutinosa* and its mechanisms of action scientifically validate its ethnopharmacological uses as a medicinal plant (Figure 3).

*J. glutinosa* has been traditionally used to treat digestive disorders. The plant’s digestive properties have been demonstrated in animal models, suggesting potential therapeutic uses for inflammatory bowel diseases, gastrointestinal problems, and motility disorders such as diarrhea [20,22]. These beneficial effects would be related to its anti-inflammatory, antioxidant, and antispasmodic properties.

The inflammation process is also related with the production ROS and the redox balance of cell and tissue, leading to cellular damage. Although there are no studies on the antioxidant activity of *J. glutinosa* in an in vivo experimental disease model, it can be suggested that its antioxidant activity may help prevent various diseases mediated by oxidative stress. Furthermore, the anti-inflammatory activity shown by *J. glutinosa* could explain its traditional use for the treatment of inflammatory pathologies of the respiratory system, such as bronchitis and asthma, rheumatoid arthritis, and muscle inflammation, as well as gastrointestinal disorders such as appendicitis.

Uncontrolled inflammatory processes in the nervous system promote the production of ROS and contribute to the development of different nervous system diseases [54]. Several studies have associated oxidative stress with the pathogenesis of Alzheimer’s disease, demonstrating that ROS promote the formation and accumulation of amyloid peptides [55]. In other nervous system disorders, such as Parkinson’s disease, Huntington’s disease, amyotrophic lateral sclerosis, and ischemic stroke, a relationship between the production of ROS and neurodegenerative processes has also been observed [56]. In *C. elegans*, the antioxidant activity of *J. glutinosa* has shown protective activity against oxidative stress and the capacity to inhibit amyloid aggregation, preventing Alzheimer’s disease.

Moreover, oxidative stress also appears to play a fundamental role in the development of depression, one of the most common psychiatric disorders worldwide [57]. The antioxidant activity and the ability to inhibit CNS enzymes such as AChE, MAO, and TYR, could explain the traditional use of *J. glutinosa* in treating disorders of the nervous system.

On the other hand, oxidative stress and inflammatory mediators are involved in pathologies of the cardiovascular system [54]. ROS can alter vascular tone and function by decreasing NO bioavailability or altering NO signaling, which are important risk factors for the development of endothelial dysfunction, the proliferation and migration of vascular cells, inflammation, and apoptosis. These processes can lead to the development of hypertension, atherosclerosis, and other cardiac pathologies. In arterial hypertension, L-type voltage-gated calcium channels play a very important role. These channels are involved in regulating the vascular tone of resistance arterioles, as well as the secretion of renin and aldosterone, and therefore play a role in blood pressure control [58]. Antagonists of L-type calcium channels, such as verapamil or nifedipine, are widely used as antihypertensive drugs. By blocking these channels, calcium entry from the extracellular medium decreases. The reduction in cytosolic calcium inhibits calcium release from the endoplasmic reticulum, producing a relaxing effect on vascular smooth muscles [59]. *J. glutinosa* inhibits these channels, which may justify its traditional use as an antihypertensive. It has also been shown to block these channels in the smooth muscle of the small intestine, which would explain its use as an antispasmodic agent. It is likely that this effect is also observed in the bronchial smooth muscle, where blocking these channels would result in bronchodilation, opening the airway and improving respiratory symptoms associated with bronchospasm [60,61].

In addition, L-type calcium channels are also present in neurons and appear to be involved in brain diseases, such as Parkinson’s disease. Therefore, the pharmacological inhibition of these channels would also have therapeutic value for the treatment of nervous pathologies [59]. This mechanism of action would also justify the use of *J. glutinosa* as a neuroprotectant.

Although there are no studies on the bioactivity of *J. glutinosa* to validate its traditional use for renal pain, kidney stone formation, or as a diuretic, its vasodilatory, antispasmodic, antioxidant, and anti-inflammatory properties could justify these uses. Several bioactive compounds found in *J. glutinosa* have shown diuretic effects, acting on different parts of the nephron by inhibiting the action of carbonic anhydrase in the proximal tubule, the Na^+^-Cl^−^ transporter in the distal tubule, and aldosterone secretion in the collecting tubule. These effects would increase the urine production volume by preventing sodium reabsorption in the renal tubules and increasing water excretion [62,63,64]. Moreover, these compounds exert anti-urolithiasis activity to prevent the crystallization, nucleation, and aggregation of crystals, which would make *J. glutinosa* useful in the treatment of urolithiasis [64,65].

The traditional use of *J. glutinosa* for weight loss and as a lipolytic agent has been supported by in vitro studies, which demonstrate that the plant inhibits the enzymes involved in glucose and lipid metabolism, exerts a lipolytic effect, and reduces the accumulation of TG and fat in adipocytes.

Finally, the use of *J. glutinosa* as a wound disinfectant could be justified by its antimicrobial activity; however, more studies are needed to demonstrate its use as a biocide.

Several limitations have been identified in fully understanding the therapeutic potential of *J. glutinosa* and its mechanisms of action. Firstly, the variety of extraction methods hinders a proper comparison on the bioactivity and phytochemistry of the samples. Secondly, and very importantly, most studies are performed with extracts instead of isolated compounds, which, in a certain way, validates the traditional use but complicates correlations between bioactivity and certain types of compounds. Thirdly, most studies have been conducted in in vitro models, highlighting the need for further in vivo studies and clinical trials to validate its efficacy and safety. Nevertheless, based on the long-standing use of this herbal remedy without reported harmful effects, we could conclude that, in general, it is safe. However, its consumption should be avoided in pregnancy and lactation due to the lack of toxicity studies, as well as in patients that develop allergy to the Asteraceae or Compositae plant family due to the presence of sesquiterpene lactones.

## 6. Conclusions

*J. glutinosa* is one of the most important plants in the folk medicine of the Iberian Peninsula, being rich in bioactive compounds such as terpenes, phenolic acid, flavonoids, and pigments. Its traditional use has been passed down through generations and is widely practiced.

This review highlights several studies demonstrating that *J. glutinosa* possesses antimicrobial, antioxidant, anti-inflammatory, spasmolytic, digestive, antihypertensive, antidiabetic, antiobesity, and neuroprotective properties. Although these studies provide scientific support for their ethnopharmacological evidence by highlighting the relationship between pharmacological activities, mechanisms of action, and traditional uses, most of them are in vitro and ex vivo studies. Therefore, further in vivo studies and clinical trials in humans are needed to confirm these bioactivities, validate its therapeutic potential, and evaluate their safety and toxicity profiles.

## Figures and Tables

**Table 1:** Traditional uses of *Jasonia glutinosa*.

System	Traditional Uses	Form of Plant Used	Reference
Digestive	Digestive, stomach ulcer, stomach pain, colic, appendicitis, emetics, antidiarrheal, whets the appetite, carminative, antispasmodic	Infusion and maceration in anisette	[1,2,4,5,6,7]
Genitourinary	Diuretic, kidney pain, depurative, kidney stones	Infusion	[1,2]
Respiratory	Colds, flu	Infusion	[1,2,8]
	Bronchitis, asthma	Infusion, vapors released during its cooking	[1,2,8]
Endocrine-metabolic	Slimming, lipolytic	Infusion	[1,2]
Musculoskeletal	Rheumatic pain, muscle inflammation, bruises	Applying a cloth soaked in the plant decoction	[1,2,9]
Integumentary	Heal and wash wounds, ulcers, and burns Disinfectant, healing agent, and anti-inflammatory for wounds	Applying a cloth soaked in the plant decoction, or macerated in alcohol, poultices from the fried or cooked leaves that are applied to wounds Ointment	[1,2]
Nervous	Headache, antidepressant, tranquilizer, nerve calmer, analgesic, dizziness	Infusion	[1,2,6,7]
Cardiovascular	Hypotensive, venotonic, and antianemic	Infusion	[1,2]

**Table 2:** Phytochemical composition of the different *Jasonia glutinosa* extracts.

Plant Drug	Extract	Extraction Method	Phytochemical Assay	Main Phytochemical Composition	Reference
Dried leaves	The essential oil and pentane extract	Distillation and ultrasound assisted maceration	GC-MS analysis	Camphor, *endo*-borneol, α-terpineol, nerolidol and T-cadinol	[10]
Fresh Leaves	The essential oil	Steam distillation	GC-MS analysis	Camphor, borneol, caryophyllene oxide, farnesol, bornyl formate, β-pinene, eucalyptol, linalool, cadinol, and spatulenol	[11]
Air-dried aerial parts	Fraction of a benzene extract	Refluxing C_6_H_6_ and chromatography of the neutral fraction on a drySi02 column	IR spectra, H NMR, and TMS analysis	Kudtdiol, α-epoxy kudtdiol, 5-epi-kudtriol, and kudtriol	[12,13]
Dried aerial parts	CH_2_C_12_ extract	MeOH maceration, partitioning between n-hexane and 5% aq. MeOH. The aq. MeOH portion was further extracted with CH_2_C_12_	1D and 2D NMR analysis	Lucinone and glutinone	[14]
Aerial parts	Acetone/water extract	Maceration	GC-MS analysis and TLC-UV	Eudesmane alcohols	[15]
Aerial parts	MeOH/H_2_O extract	Maceration	TLC-UV, HNMR, 3C-NMR and 13C-NMR-DEP	Patuletin and quercetin glucosides derivatives	[16]
Aerial parts	Butanolic extract	Maceration	TLC-UV, HNMR, 3C-NMR and 13C-NMR-DEP	Kaempferol and quercetin glucosides derivatives	[17]
Dry plant material of commercial Rock teas	MeOH and water extracts	Ultrasonic-bath maceration for MeOH extracts, and infusion for water extracts	HPLC-MS analysis	Caffeoylquinic acids, citric acid, benzoic acid, mearnsetin, quercetin, vicenin-2, rutin, kaempferol, isorhamnetin	[18]
Dried aerial parts	Ethanolic extract	Maceration	HPLC–MS analysis	Patuletin glucopyranoside	[19]
Dried leaves	Ethanolic extract	Maceration	HPLC–DAD analysis	Caffeoylquinic acids, isoferulic acid, quercetin glycosides, kaempferol, isorhamnetin, lutein, β-carotene, chlorophyll b.	[20]
Aerial parts	MeOH (70%) extract	Soxhlet	LC-MS Analysis	Caffeoylquinic acids, quercetin glycosides, kaempferol, isorhamnetin, lutein.	[21]

**Table 3:** Studies on the bioactivities of *Jasonia glutinosa*, mechanisms of action, and its related compounds.

Biological Activity	Observed Effects/Action Mechanism	Responsible Molecule
Antimicrobial Antiprotozoal Antifungal	In vitro: inhibition of *Entamoeba histolytica*, *Leishmania donovani*, *Plasmodium falciparum* [23]. In vitro: inhibition of *Rhizopus stolonifera* [24].	Sesquiterpenoids (5-epi-kudtriol) Not analyzed
Antioxidant	In vitro: positive results in DPPH [18,24,25], ABTS [18], NADH/PMS system, X/XO system [20], FRAP, ORAC [26]. Inhibition of xanthine oxidase [20]. In vivo: reduce HSP 70, increase CAT and SOD, reduce oxidation proteins [25]. Improved the survival rate and the lifespan of *C.elegans* [28].	Not analyzed Not analyzed
Anti-inflammatory	In vitro: inhibit NO and TNF-α production in LPS-stimulated murine macrophages (J774.2). Inhibit LOX-5 [20]. Increase phagocytic capacity and respiratory burst activity in leukocytes [25]. Inhibit TBX_2_-release in mouse peritoneal cells [27]. Inhibit COX-1 and PGE_2_ in mouse peritoneal cells [27]. In vivo: reduce inflammation in a murine model of colitis. Inhibit MPO, IL-6, and the expression of iNOS and COX-2 [20]. Increase peroxidase activity, IgM levels, and Il1β gene expression in fish [25].	Not analyzed Glutinone Lucinone, glutinone, 5-epikutdtriol and kutdtriol Not analyzed
Neuroprotective	In vitro: Inhibition of CNS enzymes as AChE, MAO and TYR [28]. In vivo: Decreased Aβ-induced paralysis in *C. elegans* [28].	Not analyzed Not analyzed
Antihypertensive	In vitro: Antagonist of L-type Ca^2+^ channel in A7r5 cells [19]. Ex vivo: vasorelaxant effect in aortic rings. Prevents the increase in cytosolic calcium [19].	Not analyzed Not analyzed
Anti-obesity and antidiabetic	In vitro: inhibition of α-GLU and lipase. Anti-adipogenic activity and delipidating in murine 3T3-L1 preadipocytes [26].	Not analyzed
Digestive	Ex vivo: improve dysmotility [20] and gastrointestinal transit time in a murine model of colitis [22]. Antispasmodic effect. Inhibition of L-type Ca^2+^ channels [22]. In vivo: protective effect in a murine model of colitis. Prevent symptomatology, damage, and tissue function [20].	Not analyzed Not analyzed

## Data Availability

Not applicable.

## Abbreviations

The following abbreviations are used in this manuscript:

AChE	Acetylcholinesatase
CAT	Catalase
CNS	Central Nervous System
DSS	Dextran Sulphate Sodium
FAAH	Fatty Acid Amide Hydrolase
HSP 70	Heat Shock Protein 70
HK	Head and Kidney
iNOS	Inducible Nitric Oxide Synthase
IgM	Immunoglobulin M
IL	Interleukin
LPS	Lipopolysaccharide
LTC4	Leukotriene C4
MAO	Monoamine Oxidase
MHCII	Major Histocompatibility Complex
NF-κβ	Factor Nuclear Kappa β
NO	Nitric Oxide
Nrf2	Nuclear Factor Erythroid 2
PGE2	Prostaglandin E2
ROS	Reactive oxygen Species
SOD	Superoxide Dismutase
SSZ	Sulfasalazine
TG	Triglyceride
TNF-α	Tumor Necrosis Factor alpha
TYR	Tyrosinase
TBX2	Thromboxane B2
ZO-1	Zonula Occludens-1
α-GLU	α-Glucosidase
βDEF	β-Defensines
